# Antipodal Biosecurity? Oversight of Dual Use Research in the United States and Australia

**DOI:** 10.3389/fpubh.2014.00142

**Published:** 2014-09-16

**Authors:** Frank L. Smith, Adam Kamradt-Scott

**Affiliations:** ^1^Centre for International Security Studies, University of Sydney, Sydney, NSW, Australia

**Keywords:** dual use research of concern, biosecurity, World Health Organization, United States, Australia

The creation of a virulent mousepox virus in Australia and publication of this experiment in 2001 are often argued to mark a dangerous turn in dual use research (1). After this experiment and – far more consequential – September 11 and the anthrax letters, the oversight of dual use research in the life sciences received considerable attention in the United States. We argue that the American experience provides valuable lessons for Australia, three of which are highlighted here.

First, the international community is ill-equipped to govern the life sciences. Like the United States, Australia should therefore help itself through national regulations and oversight. Second, like most special interest groups, scientists prefer self-regulation. While this may be a practical solution for scientific publications, federally funded research warrants independent review as a condition of funding. Third, in order to provide independent review, oversight should be truly multidisciplinary, including social, political, and biological expertise. A multidisciplinary approach stands the best chance of balancing the risks and rewards of dual use research.

## Think Global, Act National

The risks and rewards of dual use research have global implications. Despite repeated calls for international leadership, however, the World Health Organization (WHO) has followed rather than led member states, and it is unlikely to adopt a more assertive role. The WHO rarely issues advice on biological weapons (although it helps to monitor some smallpox research), and it said little about dual use until after the National Academies in the United States published the influential Fink Report confronting this dilemma in 2004 (2). In 2010, the WHO published its own anodyne guidance on “responsible life sciences research,” which acknowledges the “important role of WHO to lead” but then concedes to a “country-based approach” (3). Moreover, when faced with experiments that increased the transmissibility of H5N1 influenza (4, 5), the WHO sought to distance itself from any suggestion that it might assume additional responsibilities for oversight, focusing instead on “*ad hoc* solutions” to this particular controversy (6). Simply put, the WHO lacks the resources and will necessary to govern research, so states must act on their own.

Of course, the United States was never waiting for the WHO. American guidance and oversight of dual use research draws on an older system devised for recombinant DNA research that dates back to the Asilomar Conference in 1975. The Fink Report, for instance, argued that “we now need to build upon the Asilomar experience” to manage the potential misuse of biotechnology (2). This report and the National Science Advisory Board for Biodefense (NSABB) that it inspired in turn proposed incorporating oversight of dual use research into the existing system of review by Institutional Biosafety Committees and the National Institutes of Health (NIH) Recombinant DNA Advisory Committee (7).

Though less explicit, similar logic is implied in the 2012 “Policy for Oversight of Life Sciences Dual Use Research of Concern” (8), as well as the 2013 “United States Government Policy for Institutional Oversight” (9). As it now stands, U.S. policy will address 7 types of experiments of concern that were identified by the Fink Report, along with 15 select agents that are regulated through the 2001 PATRIOT Act and the 2002 Bioterrorism Act. Oversight will be accomplished through institutional and federal review of dual use research that is funded by the government, which, if fully implemented, will probably resemble the system used for recombinant DNA research.

In Australia, at least two legislative instruments apply to the dual use dilemma. First, the *National Health Security Act 2007* regulates biosafety and biosecurity standards for handling “security sensitive biological agents.” Second, the *Defence Trade Control Act 2012* applies to dual use technology, particularly through the *Defence and Strategic Goods List*. Like the American *Commerce Control List*, the Australian list designates various “materials, chemicals, micro-organisms, and toxins” as being dual use and subject to export control (10). Responsibility for research oversight in the life sciences was devolved to the National Health and Medical Research Council (NHMRC) in 2013. As “Australia’s leading expert body promoting the development and maintenance of public and individual health standards” (11), the NHMRC is now proposing a supplement to the *Australian Code for the Responsible Conduct of Research* regarding dual use or “gain of function” research (12).

## Focus on Funding

Moving forward, Australia will need to decide what steps it will take to actually oversee research of concern, and the United States still has a long way to go in implementing its evolving policies. The success of these national systems will depend on the active participation and support of the scientific community, which has special interests in how research is governed. Yet, special interests can diverge from the public interest, and this political fact has important implications for where oversight should occur and who should be involved.

Where should oversight occur? Of all the potential points of intervention, scientific communications are probably the least practical and most controversial to regulate, as illustrated by the H5N1 publishing controversy. Granted, the pre-publication review of these experiments could be considered a partial success, since they were not simply published without first evaluating the risks (as was the case in the Australian mousepox experiment and others). It is debatable, however, how much this review process affected the outcome. Plus, the surrounding controversy suggests that many scientists fear censorship more than malicious use of their research. Insisting on self-governed or unrestricted publications, scientists are quick to cry foul over the U.S. government encroaching on the norm of scientific transparency or openness.

Adherence to this norm is easy to overstate: scientists restrict information all the time, from nuclear and trade secrets to blind peer review. Nevertheless, it is impractical for the United States or Australia to restrict information that is often disseminated in different forms and venues throughout the course of research. It is far better to focus on government funding. Many life scientists depend on this funding, which gives the government significant leverage. Requiring review as a condition of funding also provides for early oversight. This may shape the trajectory of research, thereby increasing the potential benefits while reducing the risks before there are results to worry about publishing.

Tying funding to oversight is not a new idea nor is it a complete solution. But shaping research through federal funding is a relatively efficient option for the United States, especially since research of concern represents a small fraction of dual use research. For example, the NIH spends nearly $30 billion each year on more than 50,000 projects. Of these, <800 involve agents covered by the government’s new policy, and review by the NIH “designated 10 extramural and no intramural projects as dual use research of concern” (13). While NIH is not the only relevant sponsor, these numbers suggest that funding agencies can oversee research of concern without stigmatizing it or imposing an undue burden.

Attaching oversight to federal funding is potentially an even more efficient option for Australia. Compared to the United States, the Australian funding environment is more government-driven and centralized, with relatively few private, philanthropic foundations, and a smaller ecosystem of federal sponsors. As in the United States, some research will escape oversight tied to federal funding, but in Australia, both the supply of commercial research and total government support are smaller. For example, while a large fraction of Australian medical research is funded by the NHMRC, its total budget is only about $1 billion per year. It is therefore feasible to oversee research of concern through federal funding in Australia.

Unfortunately, Australia appears poised to forgo this promising option, attempting instead to abdicate government responsibility. That may be a reasonable response to the publication problem, and the prospect that it would become an offense to “publish or otherwise disseminate” listed technology under the *Defence Trade Control Act* has prompted a proposed amendment to narrow the prohibition on publication to military goods (14). However, rather than conduct any review of the research that it funds, the NHMRC looks set to delegate this responsibility to individual researchers and their home institutions through the *Australian Code for the Responsible Conduct of Research*. Institutions may establish review bodies to ensure that their researchers’ activities and publications do not breach the Code, but this overly decentralized approach stands to be burdensome to implement, difficult to enforce, and it probably fails to address core tensions between some research and the public good.

Australia would be better served by learning from the United States. This not only means establishing an advisory board like the NSABB but, perhaps more significant, also focusing on federal oversight of federally funded research. The risks – including the risk of agencies such as NHMRC looking negligent in the event of an accident or malicious use – are too great to delegate or ignore.

## Multidisciplinary Review

Finally, who should be involved with oversight? Independent review is easier said than done. Again, scientists prefer self-regulation and, when it comes to admissible expertise, the boundaries they draw around their profession often look strategic and narrow. The H5N1 publishing controversy demonstrates that even the NSABB and WHO, which supposedly represent a range of expertise, are vulnerable to the accusation that their recommendations suffer from conflicts of interest (15). Similarly, the NIH and other funding agencies risk losing public trust if their oversight systems are stacked with researchers from the same fields that they review.

Dual use research is – by definition – a social and political issue, so oversight that lacks considerable social and political expertise is suspicious. “Separating science from politics is impossible in the real world” (16), and a breadth of expertise and perspective is critical for independent review (17). So a multidisciplinary approach to oversight is best. We would include political science, given our profession, but not to the exclusion of fields ranging from sociology and history to economics and medical anthropology. Although NSABB has voting members with a background in law, for example, legal expertise is no more a substitute for political science than chemistry is for biology.

A multidisciplinary approach can also provide valuable perspectives on how to incorporate public participation into oversight. Some scientists bristle at the very idea, consistent with their special interest in self-regulation. But federal funding is tax-payer money, and so it is not outlandish to suggest that scientists should be accountable for the resources they use. Furthermore, it is hypocritical to tout the norm of scientific transparency when opposing restrictions on scientific publications and then decry more open oversight in favor of closed or cloistered peer review. A multidisciplinary approach to oversight is only one step toward adjudicating the trade-offs involved. Yet, it is a critical step, since the positive and negative externalities of dual use research extend far beyond the laboratory.

## Conflict of Interest Statement

The authors declare that the research was conducted in the absence of any commercial or financial relationships that could be construed as a potential conflict of interest.
